# A perspective from drug user organisations on ‘ECDC and EMCDDA guidance: prevention and control of infectious diseases among people who inject drugs’

**DOI:** 10.1186/1471-2334-14-S6-S10

**Published:** 2014-09-19

**Authors:** Antonio Corbacho, Manuel Rivera, Francisco Trujillo, Maite Martí, José Carbonell, Francisco Fernández, Alexandre Rosselló, Jordi Perramon, Jaume Salse, Ignacio Ramos

**Affiliations:** 1 Associació d'intervenció comunitària en drogodependències – ASAUPAM, Badalona, Santa Coloma de Gramenet, Catalonia, Spain; 2 Associació Reus Som Útils – ARSU, Reus, Catalonia, Spain; 3 Associació de Pacients Dependents d’Opiacis – APDO, Catalonia, Spain; 4 Associació Som Útils - ASUT, Barcelona, Spain; 5 Agrupa’t, Lleida, Catalonia, Spain

## 

*ECDC and EMCDDA Guidance: Prevention and Control of Infectious Diseases among People Who Inject Drugs *[1] draws on scientific evidence to identify key components of infectious disease interventions for people who inject drugs (PWID). This commentary presents an assessment of the document from the perspective of five Spanish drug user organisations. In addition to noting ways in which we think the guidance might be strengthened, we also will discuss structural factors that hinder the implementation of disease prevention measures in PWID populations.

While the following discussion of *ECDC and EMCDDA Guidance* makes it clear that more attention needs to be given to some important issues, we would like to emphasise that our intention is not to question the overall value of this document. In fact, we would be very pleased to see European countries implement the seven key intervention components that it recommends:

• clean drug injection equipment;

• vaccination;

• drug dependence treatment;

• testing for HIV, hepatitis B, hepatitis C, tuberculosis and other infections;

• infectious disease treatment;

• health promotion; and

• targeted delivery of services

The first issue we would like to highlight is the apparent lack of effort by the European Centre for Disease Prevention and Control (ECDC) and European Monitoring Centre for Drugs and Drug Addiction (EMCDDA) to involve affected communities in the development of the guidance document. While we cannot positively determine the backgrounds of all of the authors and technical advisory group members, our guess is that out of the almost 50 people named, only one is a member of our community. Not only is this not right, but surely an opportunity was missed to include input which would have improved the value of the recommendations. In general, we think that in any elaboration of drug policy, PWID should be involved from the beginning. However, we are aware that in order to achieve this aim, it is important to potentiate the empowerment of our community, which is often found living in situations of social exclusion and marginalisation, partly because of stigma and discrimination. This makes it difficult for us to acquire the basic means to participate and to have our voices heard.

A notable shortcoming that we have identified regarding the *ECDC and EMCDDA Guidance* is a lack of attention to how gender and cultural issues should be taken into account in efforts to reach and assist the full array of PWID who need services. For example, women usually are a minority of clients in drug user services. It is easy to overlook gender-specific issues that they face, such as power dynamics that lead to higher-risk sexual practices and to unsafe injecting practices imposed by male injecting partners. Migrants and cultural minorities tend to have less contact with mainstream health services and drug services, and behaviour-change interventions for these groups need to be culturally sensitive in order to be effective.

Another matter that requires greater attention, in our opinion, is the role of safe consumption rooms. The *ECDC and EMCDDA Guidance* briefly discusses safe consumption rooms (which it refers to as “supervised injecting facilities”) as one of multiple aspects of safer injecting behaviour. For many of us who do not have a place to inject or who fear the risk of overdose, safe consumption rooms provide a much-needed alternative to the streets. Therefore we recommend that safe consumption rooms be designated a key intervention along with the other key interventions named in the guidance.

In any strategic planning, communities of people who use drugs should be considered as potential care providers for our peers. We greatly appreciate the consideration given to this issue in the guidance, and we would like to comment further on two specific factors. First, the ability of some active drug users to implement prevention activities presents an important opportunity because these individuals hold a unique position. They have ongoing contact with the target population, and they can more easily reach some hidden subgroups of drug users. This interaction happens in key moments when prevention can be more effective. Secondly, there is a need to support and care for former drug users engaged in the implementation of peer interventions because of the potential impact that this work can have on their own recovery process. Former drug users need therapeutic supervision in this situation, preferably from people who are not co-workers.

Structural interventions are understandably beyond the scope of the *ECDC and EMCDDA Guidance*, which seeks to identify good practices directly related to service provision. Nonetheless we believe that it is imperative for all stakeholders involved in implementing the guidance to take into account structural barriers and to be supportive of efforts to remove these barriers.

First, there is a need to review the international conventions that criminalise the use of drugs and push PWID into illegal situations where it is more difficult for us to make use of effective prevention measures. Secondly, the ongoing stigmatization of illegal drug use limits service provision as well as hindering our access to existing services. Drug use-associated stigma also conditions the attitudes of health professionals, law enforcement personnel, and others who interact closely with PWID, making it more difficult for us to adopt the preventive measures recommended in the *ECDC and EMCDDA Guidance*. Important steps to help overcome stigma and discrimination include changing the approach to drug use from a legal to a health problem, making society understand addiction as a disease with its own complexities, and giving voice to PWID.

We would like to close by inviting readers to reflect on three questions about the *ECDC and EMCDDA Guidance*, noting that the document was published in 2011 and that some of the key interventions are supported by a solid evidence base that has been in existence for many years. First, why is there so often a lack of political will to implement these interventions? Secondly, why is their implementation in prisons so low? And finally, why is their level of implementation so different across European countries?

It would be difficult to find another at-risk population whose needs have been so widely ignored. Our community’s capacity for lobbying and advocating is still so weak that we daily encounter the denial of basic rights such as the opportunity to access these interventions. Give us voice and respect, more often and in more situations, and we are sure that this will make a difference in the prevention of drug-related harm, including harm from the transmission of hepatitis C and other bloodborne viruses. In accordance with the preceding observations, we suggest that ECDC and EMCDDA should also explore ways to consult with people who inject drugs on an ongoing basis regarding the issues that affect us.

## Competing interests

The authors declare that they have no competing interests.

## Declarations

This article has been published as part of *BMC Infectious Diseases* Volume 14 Supplement 6, 2014: Viral Hepatitis in Europe. The full contents of the supplement are available online at http://www.biomedcentral.com/bmcinfectdis/supplements/14/S6. The publication charges for this supplement were funded by AbbVie as an unrestricted grant to Rigshospitalet, the University of Copenhagen. AbbVie further funded the printing of the supplement with additional financial support from the Drug Prevention and Information Programme (DPIP) of the European Union.
